# Molecular Evolution of Human Immunodeficiency Virus Type 1 upon Transmission between Human Leukocyte Antigen Disparate Donor-Recipient Pairs

**DOI:** 10.1371/journal.pone.0002422

**Published:** 2008-06-18

**Authors:** Marjon Navis, Diana Edo Matas, Andrea Rachinger, Fransje A. Koning, Peter van Swieten, Neeltje A. Kootstra, Hanneke Schuitemaker

**Affiliations:** Department of Experimental Immunology, Sanquin Research, Landsteiner Laboratory, Center for Infectious Diseases and Immunity Amsterdam (CINIMA) at the Academic Medical Center of the University of Amsterdam, Amsterdam, the Netherlands; Institut Pasteur, France

## Abstract

**Background:**

To address evolution of HIV-1 after transmission, we studied sequence dynamics in and outside predicted epitopes of cytotoxic T lymphocytes (CTL) in subtype B HIV-1 variants that were isolated from 5 therapy-naive horizontal HLA-disparate donor-recipient pairs from the Amsterdam Cohort Studies on HIV-1 infection and AIDS.

**Methodology/Principal Findings:**

In the first weeks after transmission, the majority of donor-derived mutations in and outside donor-HLA-restricted epitopes in Gag, Env, and Nef, were preserved in the recipient. Reversion to the HIV-1 subtype B consensus sequence of mutations in- and outside donor-HLA-restricted CTL epitopes, and new mutations away from the consensus B sequence mostly within recipient-HLA-restricted epitopes, contributed equally to the early sequence changes. In the subsequent period (1–2 years) after transmission, still only a low number of both reverting and forward mutations had occurred. During subsequent long-term follow-up, sequence dynamics were dominated by forward mutations, mostly (50–85%) in recipient-HLA-restricted CTL epitopes. At the end of long-term follow-up, on average 43% of the transmitted CTL escape mutations in donor-HLA-restricted epitopes had reverted to the subtype B consensus sequence.

**Conclusions/Significance:**

The relatively high proportion of long-term preserved mutations after transmission points to a lack of back selection even in the absence of CTL pressure, which may lead to an accumulating loss of critical CTL epitopes. Our data are supportive for a continuous adaptation of HIV-1 to host immune pressures which may have implications for vaccine design.

## Introduction

CD8+ T cell responses play an important role in the control of replication of HIV in humans and of simian immunodeficiency virus (SIV) in rhesus macaques [1], [2]. In the acute phase of infection, control of HIV-1 and SIV viremia has been correlated with the appearance of virus specific CD8^+^ T cells [2]–[5] and depletion of CD8+ T cells during the chronic phase of SIV infection was associated with a rise in viral load, implicating the importance of CD8+ T cells in controlling SIV replication [5].

HIV-and SIV infection are characterized by the presence of multiple variants within individuals [6]–[9]. This diversity is a consequence of high viral turnover, high viral reverse-transcriptase (RT) error rate, recombination, and selective pressures exerted by the host's immune system, including CD8^+^ T cell responses [10]–[12]. Indeed, the generation of 10^8^–10^9^ new viral particles per day in chronically infected individuals [13], [14] creates an environment in which, in the presence of immune selection pressure exerted by CD8^+^ T cells, a large number of CD8^+^ T cell escape variants should be selected every day.

Evasion of the host CD8+ T cell responses is indeed a major factor influencing the evolution of HIV-1. The CD8+ T cell repertoire has the potential to detect many small peptide sequences encoded throughout the HIV-1 genome. Evasion of CD8+ T cell responses involves mutations within and outside targeted epitopes that can result in the inability of the peptide to bind to Class I MHC, the loss of recognition of the epitope by the CD8+ T cell receptor, or interference with peptide processing [15]–[19].

HIV-1 and SIV escape from CD8^+^ T cell recognition has been well documented in the acute and chronic phases of HIV-1 and SIV infections [15], [17], [18], [20]–[23] and in some individuals, the emergence of viral escape mutations preceded rapid disease progression [12], [23]–[25].

Transmission of viral escape variants to a new host has been documented in both horizontal and vertical HIV-1 infections [18], [26]–[30]. The persistence of CD8^+^ T cell escape variants of HIV-1 after transmission may depend on the balance between CD8^+^ T cell–mediated selective pressures and cost to viral replication fitness. Indeed, reversion to wild-type sequence will most likely occur if the escape mutation is associated with at least some replication fitness cost for the virus [31] and provided that the escape variant is transmitted to a non–HLA-matched recipient in whom similar CD8^+^ T cell selective pressures on that same epitope will not be elicited.

To date, post-transmission reversions of CTL escape mutations have been studied in the SIV macaque model [32] and for HIV-1 mainly in the highly conserved Gag region [33], [34], and in epitopes that are restricted by protective HLA-B57 alleles in the virus donor [27] or in situations where the HLA type of the donor, and thus the position of CTL escape mutations, was not known [35].

Here, we studied viral gag, env, and nef sequences of clonal HIV-1 variants that were isolated from 5 HIV-1 donors close to the moment of HIV-1 transmission and at multiple timepoints after seroconversion from their HLA disparate recipients who participate in the Amsterdam Cohort Studies on HIV infection and AIDS.

## Results

### HLA disparate donor-recipient pairs

To analyze the dynamics of potential CTL escape mutations in donor-HLA-restricted epitopes after transmission, we studied sequence changes in HIV-1 variants isolated from known HLA disparate donor-recipient pairs (Table 1 and 2). Deduced amino acid (AA) sequences from Gag (AA position 90–340), Env (gp120, AA position 80–510), and Nef (AA position 1–180) were generated from clonal virus variants that were isolated from donors and recipients at time points as closely as possible to the HIV-1 transmission event. From donor D5 HIV-1 variants were additionally isolated 28 months after transmission. From all recipients additional clonal HIV-1 variants were isolated between 9 and 22 months from a time point 54–112 months after transmission.

**Table 1:** HLA typing of donors and recipients involved in HIV-1 transmission.

Patient	Date of seroconversion (SC) or seroprevalent entry (SP) in cohort	HLA type
D1	05-08-1987 (SC)	A*01, A*24, B*07, B*07
R1	28-11-1988 (SC)	A*0201, A*3004, B*1401, B*5108^a^
D2	23-01-1985 (SP)	A*2301^a^, A*3301, B*7801, B*1503
R2	28-10-1986 (SC)	A*0201, A*1101, B*4001, B*5201
D3	04-02-1985 (SP)	A*2301^a^, A*0101, B*40, B*49^a^
R3	08-05-1987 (SC)	A*24, A*26, B*27, B*0801
D4	07-03-1988 (SP)	A*01, A*03, B*07, B*08
R4	25-09-1986 (SC)	A*3604^a^, A*0201, B*0801, B*40
D5	24-02-1988 (SP)	A*0201, A*3201, B*07, B*35
R5	05-01-1987 (SC)	A*0207, A*0207, B*0801, B*27

a Epitopes for subtypes A^*^2301, A^*^3604 and B^*^49 were not available in the Los Alamos database and therefore for these individuals only the other HLA epitopes were used for prediction of epitopes. D: donor; R: recipient

**Table 2:** Characteristics of donors and recipients involved in HIV-1 transmission

Donor	Time point of analysis^a^ (weeks)	CD4 (cells/μl)	Plasma load (log copies/ml)	Number of clones analysed	Recipient	Time point of analysis (months)	CD4 (cells/μl)	Plasma load (log copies/ml)	Number of clones analysed
D1	0	500	5.60	5	R1	0.75	670	3.00	2
						18	580	4.26^b^	10
						54	450	5.43	4
D2	−23	1100	4.46	4	R2	0.5	720	3.00^b^	2
						14.25	1150	4.67^b^	5
						112.2	100	5.71	3
D3	4	460	4.67	5	R3	0.75	590	4.52^b^	2
						9	960	3.95^b^	1
						107.5	500	3.00	2
D4	77	380	4.81	5	R4	0.75	950	5.84	5
						18	490	4.34^b^	2
						95.8	620	4.20	3
D5	102	600	4.79	5	R5	0.75	370	4.08^b^	2
	126	470	4.64	5		22	330	3.76	4
						97.8	80	4.92	4

a Weeks prior to or after seroconversion of the recipient

b Plasma load determined 3 months before the time point of virus isolation

Phylogenetic analysis of Env sequences demonstrated that HIV-1 variants from reported transmission couples grouped together in a phylogenetic tree indicating that transmission between partners was indeed highly likely (Supplementary Figure S1).

Availability of the HLA-A and –B typing from the donors and the Gag, Env, and Nef sequences from their viruses allowed an accurate estimation of AA differences within and outside predicted HLA-restricted-epitopes relative to the HIV-1 subtype B consensus sequence from the Los Alamos Database (http://www.HIV-1.lanl.gov). Amino acids that changed into a residue identical to the HIV-1 subtype B consensus for that position were considered reversions. Sequence changes away from the HIV-1 subtype B consensus, including escape mutations in predicted CTL epitopes restricted by the HLA type of the recipient, were considered forward mutations.

Epitopes for subtype A*2301 (expressed by donors D2 and D3), B*49 (also expressed by donor D3), and A*3604 (expressed by recipient R4) were not available in the Los Alamos Database. To avoid an overestimation of the number of epitopes in the viruses from these donors we only used their other HLA types for the prediction of CTL epitopes.

### Reversion of mutations towards the HIV-1 subtype B consensus sequence upon viral transmission between HLA disparate donor-recipient pairs

**Figure 1:**
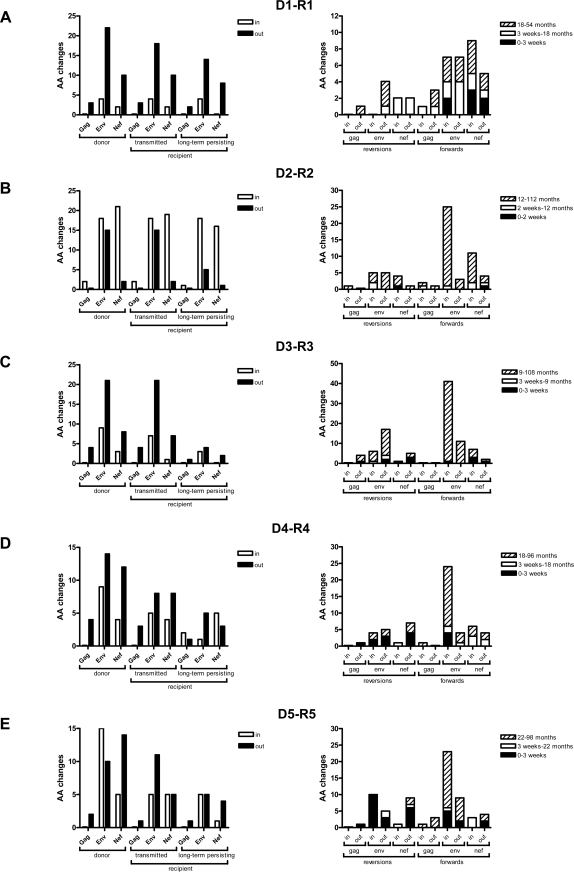
Absolute number of AA differences relative to the consensus HIV-1 subtype B sequence in HIV-1 Gag, Env and Nef from 5 donor-recipient pairs (a–e). Left panels: Based on the HLA types of donors we determined if AA differences were inside (white bars) or outside (black bars) predicted CTL epitopes. We distinguished AA differences that were present in the donor (donor), that were still present early after transmission to the recipient (transmitted) and that were still present in recipient viruses after long-term follow-up (long-term persisting) (a–e left panel). Right panels: Based on the HLA types of the donors, we determined AA residues that were lost in the recipient immediately after transmission (reversions after 2–3 weeks, black stacks), that reverted during the first years after SC (reversions after 9–22 months, white stacks), or that had reverted by the end of follow-up (reversions after 54–112.2 months, hatched stacks). In predicted recipient-HLA-restricted epitopes the number of mutations was determined directly after SC (forwards after 2–3 weeks, black stacks), during the first years (forwards after 9–22 months, white stacks), or after long-term follow-up (forwards after 54–112.2 months, hatched stacks). *In* and *out* refer to mutations inside and outside predicted epitopes restricted by donor-HLA in the category “reversions” and by recipient-HLA in the category “forwards”.

First we calculated the number of AA differences relative to the HIV-1 subtype B consensus sequence in clonal HIV-1 variants isolated from the donors at a time point as closely as possible to the transmission event. Mutations in predicted epitopes that were restricted by the donor HLA type were considered potential CTL escape mutations (Fig 1, left panels, donor). The earliest recipient viruses were then studied for preservation of AA differences, within or outside predicted donor-HLA restricted-epitopes, that we had first identified in the donor viruses (Figure 1, left panels, recipient). The majority of AA differences that were present in HIV-1 variants from the donor were still present in HIV-1 variants that were isolated from the recipient within 2–3 weeks after the transmission event (Figure 1, left panels, “transmitted”).

Amino acid differences relative to the consensus B sequence, that were present in donor viruses, but absent in the earliest recipient viruses were considered to have reverted within the first 2–3 weeks after transmission. In HIV-1 variants from donors D1–D5, we observed a total of respectively 41, 58, 45, 43, and 46 AA differences in Gag, Env, and Nef, relative to the HIV-1 subtype B consensus sequence for these genes (Figure 1, left panel). Only 1, 5, 0, 2 and 4 AA changes, respectively per donor, were at anchor residue positions (Supplementary Table S1).

Of all AA differences in Gag, Env, and Nef, only 0, 1, 7, 10, or 20, respectively, had reverted in viruses isolated from the recipients early after transmission and less than 50% of these reversions were in predicted epitopes restricted by the HLA type of the donor (Figure 1, right panels, black stacks).

In HIV-1 variants isolated from all recipients 9–22 months after the transmission event, a limited number of additional reversions had occurred, again both in- and outside predicted donor-HLA-restricted epitopes (Figure 1, right panels, white stacks).

At the end of follow-up (54–112 months after transmission) HIV-1 variants isolated from recipients R1 to R5 revealed respectively 4, 13, 23, 7, and 2 additional reversions of which only 0, 6, 5, 2 and 0 were in predicted donor-HLA-restricted epitopes (Figure 1, right panels, hatched stacks).

Amino acid differences relative to the consensus B sequence that were present in donor viruses, and still present in recipient viruses isolated at the end of follow-up (54–112 months after transmission), were considered long-term persisting AA differences (Figure 1, left panels, recipient).

Overall, the number of reversions was low and predominantly outside predicted CTL epitopes restricted by the HLA type of the donor. The exact AA residues within predicted CTL epitopes that are restricted by the donor-HLA type and the AA residues that reverted to the HIV-1 subtype B consensus sequence in viruses isolated from the recipient are shown in Supplementary Table S1.

### Forward mutations in HIV-1 in recently infected individuals

Next we calculated the number of forward mutations that occurred within and outside predicted CTL epitopes restricted by the HLA-type of the recipients, in viral sequences from clonal HIV-1 variants that were isolated at relatively early, intermediate, and late time points after transmission from all recipients (Figure 1, right panels).

During the first 2–3 weeks after transmission, viruses from recipients R1 to R5 showed 7, 1, 5, 4, and 9 forward mutations, the majority of which in predicted recipient-HLA-restricted CTL epitopesb (Figure 1, right panel, black stacks).

In HIV-1 variants that were isolated at the intermediate time point (range 9 to 22 months after transmission) from R1-R5, a total of 11, 5, 0, 8 and 4 forward mutations had accumulated, the majority of which again in predicted recipient-HLA-restricted CTL epitopes.

At the end of follow-up (54–112 months after transmission), a total of 14, 40, 56, 28 and 30 forward mutations had accumulated in HIV-1 variants from recipients R1 to R5 respectively. Of these mutations, the majority (7, 34, 44, 22, and 18, respectively for viruses from R1–R5) were in predicted recipient-HLA-restricted epitopes, of which respectively 2, 10, 12, 6 and 8 were at anchor residue positions. An overview of the exact AA residues in predicted CTL epitopes restricted by the recipient's HLA types that mutated away from the HIV-1 subtype B consensus sequence in viruses isolated from the recipient is shown in Supplementary Table S1.

### Dynamics of HIV-1 sequences upon transmission between HLA disparate donor-recipient pairs

HIV-1 virus variants from all donors showed the lowest number of AA differences with the subtype B consensus in Gag and the highest number of differences in Env, confirming their respective highly conserved and variable nature. A similar observation was made in HIV-1 variants from all recipients in which the number of forward mutations was the highest in Env and Nef.

In summary, the donors of our 5 horizontal transmission pairs harbored HIV-1 variants that contained a total of 233 AA differences relative to the HIV-1 subtype B sequence, of which 93 (40%) AA differences were in predicted donor-HLA-restricted epitopes. In HIV-1 variants isolated from the recipients early after the transmission event, a total of 188 AA (81%) differences were still present of which 72 (38%) were in epitopes restricted by the HLA type of the donor. Of the 20 AA differences that had rapidly reverted in the recipient, 14 (70%) were in donor-HLA-restricted epitopes and equally distributed over Gag, Env, and Nef genes.

Throughout the subsequent follow-up period, which varied from 9 to 22 months after transmission between recipients, we found similar ratios of reverting and forward mutations, although total numbers of AA changes were low (Ratio reversion/forward mutations for R1: 5/11; R2: 2/5; R3: 3/0; R4: 1/8; R5: 4/4). During the subsequent follow-up period of 54–112 months after transmission, sequence evolution in HIV-1 variants from all recipients was dominated by forward mutations (Ratio reversion/forward mutations R1: 4/14; R2: 13/39; R3: 23/56; R4: 7/27; R5: 2/29). In this later phase of infection, 54–100% of reversions were outside predicted donor-HLA-restricted epitopes, while the majority of all forward mutations (50–85%) had occurred inside predicted recipient-HLA-restricted epitopes.

### Dynamics of HIV-1 RNA in plasma as compared to biological cloned HIV-1 variants

Finally, we analyzed whether the sequence dynamics as observed in the longitudinally isolated clonal HIV-1 variants were representative for HIV-1 sequence changes in plasma. To this end we compared sequences of the Env V3V4 region of the clonal HIV-1 variants of all recipients with sequences from the V3V4 region from HIV-1 RNA in plasma from the same or similar time points.

Confirming the close relation between the viral quasispecies in plasma and in productively infected cells, all reversions and forward mutations that had been observed in- and outside predicted CTL epitopes in V3V4 of the clonal HIV-1 variants were also present in viral RNA sequences from plasma (data not shown). The identical sequence dynamics in the V3V4 Env region of the clonal HIV-1 variants studied here and in the viral RNA in plasma suggest that the sequence dynamics observed in our longitudinally obtained clonal HIV-1 variants are a true reflection of the sequence dynamics in vivo.

## Discussion

In this study, we analyzed sequence evolution of HIV-1 in 5 recipients of HLA-disparate HIV-1 transmission pairs that participate in the Amsterdam Cohort Studies on HIV-1 infection and AIDS. We isolated multiple clonal HIV-1 variants from productively infected cells throughout the course of infection and analyzed sequence evolution in Gag, Env and Nef. This allowed us not only to study virus evolution in recipients and their donors, but also to compare sequence evolution in different genes.

In agreement with a recent study that also focused on viral evolution in the first months to years after HIV-1 infection [35], we observed a considerable number of mutations already very early after seroconversion. In that study, however, early sequence evolution was dominated by reversions while in our study [35], reversions and forward mutations contributed equally to the early sequence dynamics in HIV-1.

In analogy to previous studies [28], [35], we determined sequence differences relative to the HIV-1 subtype B consensus sequence from the LANL HIV Sequence Database {REF}. Furthermore, availability of sequences from both donor and recipient virus populations allowed us to accurately calculate the number of transmitted AA differences that subsequently reverted in recipient viruses, both in and outside CTL epitopes restricted by the HLA-type of the donor. We used predicted epitopes rather than epitopes for which CTL reactivity was actually demonstrated [36] as to prevent a bias in our analyses towards better investigated HIV-1 genes (Gag) and HLA types (HLA A*02, B*57 and B*27). The observation that for the vast majority of predicted epitopes CTL recognition has indeed been demonstrated [37] supports our approach. Moreover, when we based our epitope mapping on a recent comprehensive collection of reported epitopes by Frahm and Brander [36], similar to the approach of Li et al [35], we still observed a similar contribution of forward and reverting mutations to the early sequence dynamics (data not shown). The limit of this latter approach, however, is that a much lower number of mutations can be interpreted as potential CTL escape mutation.

Based on the observation that CTL escape mutations revert upon transmission [27], [32], [35] Leslie et al. were the first to conclude that this may be driven by a gain of fitness, implying that at least some CTL escape mutations come at a substantial fitness cost [27]. They monitored the T242N mutation in the HLA-B57 restricted TW10 epitope during mother-to-child transmission. The N242T reversion was observed when the virus was transmitted from an HLA-B57 positive mother to an HLA-B57 negative child while the 242N residue was conserved when the virus was transmitted to an HLA-B57 matched child. In agreement with the hypothesis that reversion of mutations is driven by gain of fitness, Li et al. observed that reverting mutations preferentially arose within highly conserved residues and suggested that the severity of fitness loss associated with CTL escape mutations, so the strength of back selection, determines the kinetics by which escape mutations and reversions occur [38]. A recent study has shown that non-transmission or reversion after transmission was associated with reduced fitness thereby in support of the notion that some escape mutations come at a fitness cost. However, that study again only focused on the highly conserved p17 and p24 in Gag [39].

In our study, even the limited number of very early reversions were not restricted to highly conserved regions but equally distributed in Gag and Env although the number of mutations in Gag in donor virused was low. It cannot be excluded that the donor virus population in the study by Li et al. had substantially more mutations in the conserved Gag region which could relate to the HLA type of the donor. Unfortunately, this information was not available as in that study virus donors were not known. Nevertheless, if rapid reversion of mutations is considered to reflect the severity of the fitness cost associated with these mutations, our data seem to suggest that CTL escape mutations outside conserved regions may also be associated with a severe fitness cost to the virus.

Our sequence analysis was performed on clonal HIV-1 variants isolated from single productively infected cells as this allows the comparison of sequence dynamics in different genes of a single virus variant. Even though clonal virus isolation does not suffer from the competitive selection bias of bulk cultures, a point of concern of working with cultured viruses is that the observed AA reversions may not have occurred in the recipient but during the virus isolation procedure. However, a 5 months culture of 2 donor and 3 recipient virus variants in 96 replicates per virus, resulted in a maximum of only 2 random nucleotide changes in the V3/V4 region in 50 to 100% of the microcultures per virus variant (data not shown), indicating that it is highly unlikely that during the short term culture for virus isolation any reversions have occurred.

Another concern may be that these clonal HIV-1 variants may not be fully representative of the total, replication competent viral quasispecies in plasma. However, AA changes in the Env V3V4 region that we had observed over time in clonal HIV-1 variants were identical to AA changes in the V3V4 region in viral RNA in plasma from the same individuals. Moreover, phylogenetic analysis of env sequences shows that the viral quasispecies in plasma and isolated replication competent clonal HIV-1 variants from similar time points from the same individual are very closely related (Navis et al. manuscript in preparation). Finally, it has been shown that the kinetics of viral load changes and the emergence of drug resistance mutations in plasma/serum and productively infected cells are highly correlated [40]–[42].

During the intermediate follow-up period that varied from 9 to 22 months after transmission between recipients, we found only one reverting mutation and very few forward mutations in Gag while in Env and Nef the numbers of AA reversions and forward mutations were somewhat higher albeit still low. Only during the last follow-up period (54–112 months after transmission) sequence evolution was dominated by forward mutations that mainly occurred in epitopes restricted by recipient HLA. Interestingly, in 4 of 5 recipients, the proportion of forward mutations in Env was much higher within than outside predicted CTL epitopes indicating that CTL pressure in Env is stronger than other selections pressures at that stage of infection. Moreover, reversions constituted nearly half of all AA differences in regions outside donor-HLA-restricted epitopes. It is tempting to speculate that these reversions involve AA residues that were selected in the donor to compensate for loss of fitness associated with CTL escape mutations [27], [43]–[46]. With reversion of the CTL mutations in the recipient, apparently some of these compensatory mutations give a fitness cost themselves, driving their reversion.

At the end of long-term follow-up, on average 43% of the transmitted CTL escape mutations in donor-HLA-restricted epitopes had reverted to the consensus sequence in viruses isolated from the recipient. Although our data confirm that intrapatient viral evolution driven by CTL pressure does not necessarily translate to the evolution of HIV-1 at the population level, more then half of the AA differences that originally occurred in the donor were still preserved later in the course of infection in the recipient, in agreement with a previous study [43].

The reversion of escape mutations in epitopes in less conserved regions of the virus is in line with many studies that have shown the presence of CTL directed against those regions [47]–[49] and with previous studies that have shown evolution towards an ancestral, or consensus sequence, upon transmission to a new host [35], [50]. Had these epitopes been permanently negatively selected, these CTL could not have been elicited in later years of the HIV-1 pandemic. The only slow reversion of mutations in the phase of infection when recipient CTL are already elicited confirm that a vaccine should not be based on the HIV-1 consensus sequence but rather should take into account all possible variation in a given epitope. Fortunately, this variation may be more limited than previously assumed [51] which may make it feasible to design a vaccine capable of eliciting effective HIV-1 specific cellular immune responses.

## Materials and Methods

### Patients

Five HIV-1 donor-recipient pairs were selected for this study. Donor-recipient pairs ACH18814-ACH18766 (donor 1 (D1)-recipient 1 (R1)), ACH19545-ACH18860 (D2-R2), ACH19500-ACH18829 (D3-R3) participated in the Amsterdam Cohort Studies on HIV-1 infection and AIDS (http://www.amsterdamcohortstudies.org) and entered the cohort studies with a discordant serostatus for HIV-1 antibodies. HIV-1 transmission occurred during active follow-up. From donor-recipient pairs ACH11686 (D4)-ACH19342 (R4) and ACH13994 (D5)-ACH18839 (R5), initially only the recipients participated in the cohort studies and seroconverted for HIV-1 antibodies during active follow-up. Their HIV-1-positive sexual partners were asked to participate in the cohort studies after the HIV-1 transmission event. Recipients R1, R2, and R5 progressed to AIDS after an asymptomatic follow-up of 73, 112, and 72 months, respectively. Recipients R3 and R4 remained asymptomatic during the total follow-up period of 157 and 148 months, respectively.

The Amsterdam Cohort Studies are conducted in accordance with the ethical principles set out in the declaration of Helsinki and written consent was obtained prior to data collection. The study was approved by the Academic Medical Center institutional medical ethics committee.

### HLA typing

Genotyping at HLA class I loci was performed by sequence specific primers (SSP) PCR as described elsewhere [52].

### Isolation of clonal HIV-1 variants

Clonal HIV-1 variants from single productively infected cells were obtained by cocultivation of serial dilutions of PBMC that were obtained around the moment of HIV-1 transmission from both the donor and the recipient with 2–3 day phytohemagglutinin stimulated PBMC from a healthy donor (PHA-PBMC) as described previously [53]. To obtain PHA-PBMC, PBMC from a healthy donor were cultured in IMDM supplemented with 10% FCS (Hyclone), 1 μg/ml PHA (Welcome), Pen/Strep (Gibco Brl), 5 μg/ml Ciprofloxacin (Bayer) for 2–3 days in a culture flask at a cell density of 5×10^6^/ml. Clonal virus variants were isolated by cocultivation of 10,000–40,000 patient PBMC with 10^5^ PHA-PBMC in a final volume of 150 μl IMDM supplemented with 10% FCS (Hyclone), Pen/Strep (Gibco Brl), 10 U/ml rIL-2 (proleukin; Chiron Benelux BV), 5 μg/ml Ciprofloxacin (Bayer) and 5 μg/ml polybrene (Sigma) for 35 days in a 96-well flat-bottom microtiter plate. Every week, culture supernatants were tested for virus production in an in-house Gag p24 antigen capture enzyme-linked immunosorbent assay. At the same time, one-third of the culture volume was transferred to new 96-well plate and fresh PHA-stimulated healthy donor PBMC were added to propagate the culture. If less than 1/3 of the microcultures per patient-PBMC dilution were positive for p24 production, cultures were considered to be infected by progeny of a single HIV infected cell. A maximum of 10 clonal virus variants were expanded by cocultivation of the cells from the microculture with 5×10^6^ PHA-PBMC at a density of 1×10^6^/ml IMDM supplemented with 10% FCS (Hyclone), Pen/Strep (Gibco Brl), 10 U/ml rIL-2 (proleukin; Chiron Benelux BV), 5 μg/ml Ciprofloxacin (Bayer) and 5 μg/ml polybrene (Sigma) in a culture flask.

### DNA isolation, PCR amplification and sequencing

Total DNA was isolated from PBMC infected with clonal HIV-1 isolates using the L6 isolation method [54]. Gag was amplified using a nested polymerase chain reaction (PCR) with outer primers Gag-forward (fw) (5′-CGACGCAGGACTCGGCTTGCTG-3′) and Gag-outer-reversed (rev) (5′-GCCTGTCTCTCAGTAC-3′) and 2 different sets of inner primers: Gag-BssHII-fw (5′-TGCTGAAGCGCCCGCACGGC-3′) or Gag-ClaI-fw (5′-GGGAGAATTAGATCGATGGG-3′) in combination with Gag-p17-rev (5′-CAAAACTCTTGCCTTATGG-3′) and Gag-p17-fw (5′-TGCTAAACACAGTGGGGGGACAT-3′) in combination with Gag-ApaI-rev (5′-TTCCTAGGGGCCCTGCAA-3′). Nef was amplified using a nested PCR with outer primers Nef-1-fw (5′-AGCCATAGCAGTAGCTGAGG-3′) and Nef-1-rev (5′-GCTTATATGCAGGATCTGAGG-3′) and inner primers Nef-2-fw (5′-AGCTTGTAGAGCTATTCGCCACA-3′) and Nef-2-rev (5′-AGCAAGCTCGATGTCAGCAG-3′). Gag and Nef PCRs were performed using Promega Taq polymerase in the presence of 2mM MgCl_2_ using the following amplification cycles: 2 min 95°C, 35 cycles of 30s 95°C, 30s 55°C, 2 min 72°C, followed by a 10 min extension at 72°C and subsequent cooling to 4°C.

Env was amplified using a nested PCR. The primary PCR was perform with forward primer TB3 (5′-GGCCTTATTAGGACACATAGTTAGCC-3′) and reverse primer TBC (5′-GCTGCCTTGTAAGTCATTGGTCTTAAAGG-3′) using the expand high fidelity Taq polymerase kit (Roche) and the following amplification cycles: 2 min 30s 94°C, 9 cycles of 15s 94°C, 45s 50°C, 2 min 72°C, 35 cycles of 15s 94°C, 45s 53°C, 2 min 72°C, followed by a 10 min extension at 72°C and subsequent cooling to 4°C. Nested PCR was performed with 3 different sets of: seq1 (5′-TACATAATGTTTGGGCCACACATGCC-3′) and seq4 (5′-CTTGTATTGTTGTTGGGTCTTGTAC-3′); seq5 (5′-GTCAACTCAACTGCTGTTAAATGGC-3′) and seq6 (5′-ATCTAATTTGTCCACTGATGGGAGG-3′); PSCfw (5′-ATCCTCAGGAGGGGACCCAGA-3′) and PSH (5′-CCATAGTGCTTCCTGCTGCT-3′). Nested PCRs were performed using Promega Taq polymerase in the presence of 2mM MgCl_2_ using the following amplification cycles: 2 min 95°C, 35 cycles of 30s 95°C, 30s 55°C, 2 min 72°C, followed by a 10 min extension at 72°C and subsequent cooling to 4°C.

PCR products were purified using EXOSAP-IT (USB, Cleveland, Ohio, USA ) and sequenced using ABI prism Big Dye Terminator v1.1 Cyclesequencing Kit (Applied Biosystems) using the nested PCR primers. Sequences were analyzed on the Applied Biosystems/Hitachi 3130 xl Genetic Analyzer.

### HIV-1 RNA isolation from plasma, cDNA synthesis and sequencing

From all recipients, plasma samples were available close to the early (range 0–2 months later) and intermediate (range 12 month) time points. From recipient R1 an additional plasma sample of the late timepoint (54 months post SC) was available. Viral RNA was isolated from plasma or serum using the QIAgen Viral RNA Mini Kit and reverse transcribed into cDNA with Superscript II RnaseH Reverse Transcriptase (Invitrogen) using outer primer seq2 (5′-TCCCTCATATCTCCTCCTCCAGGTC-3′). cDNA from the V3-V4 *env* region, derived from viral RNA in patient plasma was amplified using nested PCR with the following primer combinations: outer primers seq2 (5′-TCCCTCATATCTCCTCCTCCAGGTC-3′) and seq3 (5′-TATGGGATCAAAGCCTAAAGCCATG-3′), inner primers seq5 (5′-GTCAACTCAACTGCTGTTAAATGGC-3′) and seq6 (5′-ATCTAATTTGTCCACTGATGGGAGG-3′). PCRs were performed using the following amplification cycles: 5 min 94°C, 35 cycles of 45s 94°C, 30s 50°C, 90s 72°C, followed by a 6 min extention at 72°C and subsequent cooling to 4°C. Bulk PCR products resulting from plasma RNA were cloned in the pGEM-Teasy Vector system (Promega) and transformed into DH5α competent cells (invitrogen). The V3-V4 insert was amplified by PCR using primer pair seq5-seq6. PCR products were purified using EXOSAP-IT (USB, Cleveland, Ohio, USA ) and sequenced using the ABI prism Big Dye Terminator v1.1 Cyclesequencing Kit (Applied Biosystems) using the nested PCR primers. Sequences were analyzed on the Applied Biosystems/Hitachi 3130 xl Genetic Analyzer.

### Phylogenetic analysis

Sequences of env gp120 were manually aligned using ClustalW included in the software package BioEdit [55] (BioEdit v 7.0.5, Tom Hall, Ibis Therapeutics, Carlsbad, CA). The matrix of the aligned sequences was imported into the tree building software PAUP* [56] (http://paup.csit.fsu.edu/), and an initial neighbour-joining (NJ) tree [57] was reconstructed under the Hasegawa-Kishino-Yano (HKY85) model of evolution [58]. A heuristic search for a maximum-likelihood tree, under time reversible model of nucleotide substitution, with proportion of invariable sites and gamma rate distribution was made. The robustness of the NJ phylogeny was assessed by bootstrap analysis with 1,000 rounds of replication.

### Prediction of CTL epitopes

Epitopes were predicted using motifscan in the Los Alamos Database [59] in which deduced amino acid Gag, Env, and Nef sequences were scanned for potential epitopes based on HLA binding motifs (http://www.HIV-1.lanl.gov/).

### Sequence analysis

Amino acid changes towards the consensus sequence of HIV-1 subtype B were considered reversions. Sequence changes away from the subtype B HIV-1 consensus sequence, including escape mutations in predicted CTL epitopes restricted by the HLA type of the recipient, were considered forward mutations. When 4 or more clonal HIV-1 variants from a single time point were available for analysis, a reversion or forward mutations was counted when the mutation was present in 50% or more of the clonal HIV-1 variants. When less then 4 clonal HIV-1 variants were isolated from a single time point during infection, an AA change was considered a reversion or forward mutation only when present in all viruses. The number of clonal HIV-1 variants that were analysed per individual per timepoint is indicated in Table 2.

## Supporting Information

Table S1 CTL escape mutations and reversions in HIV-1 variants isolated from HLA disparate pairs.
(0.53 MB DOC)

Figure S1 Phylogenetic analysis of env sequences of clonal HIV-1 variants isolated from donors (D1-5) and recipients (R1-5) involved in homosexual HIV-1 transmission. Shown is a maximum likelihood tree with bootstrap values obtained from neighbor joining analysis. Bootstrap values are given and show that HIV-1 variants from donors and recipients are related.
(0.35 MB TIF)
