# Supplementary material for: Molecular Evolution of Human Immunodeficiency Virus Type 1 upon Transmission between Human Leukocyte Antigen Disparate Donor-Recipient Pairs
Source: PLoS One. 2008 Jun 18;3(6):e2422. doi: 10.1371/journal.pone.0002422 (PMC2409968; doi:10.1371/journal.pone.0002422)
Supplement: Table S1 — CTL escape mutations and reversions in HIV-1 variants isolated from HLA disparate pairs. (0.53 MB DOC) [file pone.0002422.s001.doc]

**Table SI** CTL escape mutations and reversions in HIV-1 variants isolated from HLA disparate pairs

|  | **Gag** |  |  |  | **Nef** |  |  |  | **Env** |  |  |  |
| --- | --- | --- | --- | --- | --- | --- | --- | --- | --- | --- | --- | --- |
| **D1** | A*01 | A*24 | B*07 | B*07 | A*01 | A*24 | B*07 | B*07 | A*01 | A*24 | B*07 | B*07 |
|  |  |  |  |  |  |  | R71K |  |  |  | V83I |  |
|  |  |  |  |  |  |  | Y135F |  |  |  | V84E |  |
|  |  |  |  |  |  |  |  |  |  |  | **L85V** |  |
|  |  |  |  |  |  |  |  |  |  |  | V172L |  |
|  |  |  |  |  |  |  |  |  |  |  | E329D |  |
| **reverting mutations in R1** |  |  |  |  |  |  |  |  |  |  |  |  |
| **t1** |  |  |  |  |  |  |  |  |  |  |  |  |
| **t2** |  |  |  |  |  |  | K71R |  |  |  |  |  |
|  |  |  |  |  |  |  | F135Y |  |  |  |  |  |
| **t3** |  |  |  |  |  |  |  |  |  |  |  |  |
|  |  |  |  |  |  |  |  |  |  |  |  |  |
| **forward mutations in R1** | A*0201 | A*3004 | B*1401 | B*5108^a^ | A*0201 | A*3004 | B*1401 | B*5108^a^ | A*0201 | A*3004 | B*1401 | B*5108^a^ |
| **t1** |  |  |  |  |  | A53T |  |  |  | R291K |  |  |
|  |  |  |  |  |  | C55S/I |  |  |  | R343G |  |  |
|  |  |  |  |  |  | **Y120F** |  |  |  |  |  |  |
| **t2** |  | K302R |  |  |  | L85F |  |  |  | A344T/I |  |  |
|  |  |  |  |  |  | H89Y/F |  |  | K355E/T |  |  |  |
| **t3** |  |  |  |  |  | P14S |  |  |  | V83M |  |  |
|  |  |  |  |  |  | V16I |  |  |  | T167S |  |  |
|  |  |  |  |  |  | **Y102H** |  |  |  | R322K |  |  |
|  |  |  |  |  |  | S163C |  |  |  |  |  |  |

**Table SI** Continued

|  | **Gag** |  |  |  | **Nef** |  |  |  | **Env** |  |  |  |
| --- | --- | --- | --- | --- | --- | --- | --- | --- | --- | --- | --- | --- |
| **D2** | A*2301^a^ | A*3301 | B*7801 | B*1503 | A*2301^a^ | A*3301 | B*7801 | B*1503 | A*2301^a^ | A*3301 | B*7801 | B*1503 |
|  |  |  | I223N |  |  |  | V10G |  |  |  | V84A |  |
|  |  |  | E312D |  |  |  | V11G |  |  |  |  | V172M |
|  |  |  |  |  |  |  | P14A |  |  |  |  | E175D |
|  |  |  |  |  |  |  | T15A |  |  |  | K181R |  |
|  |  |  |  |  |  |  | R22Q |  |  |  | D188G |  |
|  |  |  |  |  |  |  | **A23Q** |  |  |  | R201T |  |
|  |  |  |  |  |  |  | V33A |  |  |  | **A228T** |  |
|  |  |  |  |  |  |  | K39A |  |  |  | N238K |  |
|  |  |  |  |  |  |  | H40R |  |  |  | T249K |  |
|  |  |  |  |  |  |  | S45I |  |  |  | E277G |  |
|  |  |  |  |  |  |  | S46N |  |  |  | E278D |  |
|  |  |  |  |  |  |  | **A50T** |  |  |  | N309H |  |
|  |  |  |  |  |  |  | E65D | E65D |  |  | I341L |  |
|  |  |  |  |  |  |  | Y102H |  |  |  | **R343S** |  |
|  |  |  |  |  |  |  | F143Y |  |  |  | A344V |  |
|  |  |  |  |  |  |  | K152Q |  |  |  | T394S |  |
|  |  |  |  |  |  |  | E155K |  |  |  | **G406N** |  |
|  |  |  |  |  |  |  | M168I |  |  |  | R446S |  |
|  |  |  |  |  |  |  | S163C |  |  |  |  |  |
|  |  |  |  |  |  |  | L170Q |  |  |  |  |  |
|  |  |  |  |  |  |  | V182I/M | V182I/M |  |  |  |  |
|  |  |  |  |  |  |  |  |  |  |  |  |  |
| **reverting mutations in R2** |  |  |  |  |  |  |  |  |  |  |  |  |
| **t1** |  |  |  |  |  |  | Y143F |  |  |  |  |  |
| **t2** |  |  |  |  |  |  |  |  |  |  | G188D |  |
|  |  |  |  |  |  |  |  |  |  |  | S394T |  |
| **t3** |  |  | I223N |  |  |  | A15T |  |  |  |  | M172V |
|  |  |  |  |  |  |  | N46S |  |  |  |  | D175E |
|  |  |  |  |  |  |  | M/I182V |  |  |  | H309N |  |

**Table SI** Continued

|  | **Gag** |  |  |  | **Nef** |  |  |  | **Env** |  |  |  |
| --- | --- | --- | --- | --- | --- | --- | --- | --- | --- | --- | --- | --- |
| **forward mutations in R2** | A*0201 | A*1101 | B*4001 | B*5201 | A*0201 | A*1101 | B*4001 | B*5201 | A*0201 | A*1101 | B*4001 | B*5201 |
| **t1** |  |  |  |  |  |  |  |  |  |  |  |  |
| **t2** | T280A/S |  |  |  |  |  |  | **I43M/L** |  |  |  | T292P |
|  |  |  |  |  |  | V153I |  |  |  |  |  |  |
| **t3** |  | R286K |  |  |  | S9I |  |  |  |  |  | N98S |
|  |  |  |  |  |  | P14E |  |  |  | I157M |  |  |
|  |  |  |  |  |  | V16I |  |  |  | Q173K |  |  |
|  |  |  |  |  |  | **K39T** |  |  |  | N238R |  |  |
|  |  |  |  |  |  | K92R | K92R |  |  | **K241Q** |  |  |
|  |  |  |  |  |  | E93D | **E93D** |  |  | R282K |  |  |
|  |  |  |  |  |  | V148L |  | **V148L** |  | T287S |  | T287S |
|  |  |  |  |  |  | **K152E** |  |  |  | I316V |  |  |
|  |  |  |  |  | R178K | R178K |  |  |  | H317R |  |  |
|  |  |  |  |  |  |  |  |  |  | **R322S** |  |  |
|  |  |  |  |  |  |  |  |  |  |  |  | F324I |
|  |  |  |  |  |  |  |  |  |  | E329D |  | E329D |
|  |  |  |  |  |  |  |  |  |  | D333N |  |  |
|  |  |  |  |  |  |  |  |  |  | **K345E** |  |  |
|  |  |  |  |  |  |  |  |  |  | E359Q |  |  |
|  |  |  |  |  |  |  |  |  |  | V367E |  | **V367E** |
|  |  |  |  |  |  |  |  |  |  | N369K |  |  |
|  |  |  |  |  |  |  |  |  |  | N412K |  |  |
|  |  |  |  |  |  |  |  |  |  | R425K |  | R425K |
|  |  |  |  |  |  |  |  |  |  | R450K |  |  |
|  |  |  |  |  |  |  |  |  |  | L459I |  |  |
|  |  |  |  |  |  |  |  |  |  | E468Q |  |  |
|  |  |  |  |  |  |  |  |  |  | K497K |  |  |
|  |  |  |  |  |  |  |  |  |  | **K507Q** |  | K507Q |

**Table SI** Continued

|  | **Gag** |  |  |  | **Nef** |  |  |  | **Env** |  |  |  |
| --- | --- | --- | --- | --- | --- | --- | --- | --- | --- | --- | --- | --- |
| **D3** | A*2301^a^ | A*0101 | B*40 | B*49^a^ | A*2301^a^ | A*0101 | B*40 | B*49^a^ | A*2301^a^ | A*0101 | B*40 | B*49^a^ |
|  |  |  |  |  |  |  | D28E |  | A290T |  |  |  |
|  |  |  |  |  |  |  | E98D |  | T292I |  |  |  |
|  |  |  |  |  |  |  |  |  |  |  | N347E |  |
|  |  |  |  |  |  |  |  |  |  |  | Q352K |  |
|  |  |  |  |  |  |  |  |  |  |  | V354A |  |
|  |  |  |  |  |  |  |  |  | G362E |  |  |  |
|  |  |  |  |  |  |  |  |  | K364A |  |  |  |
|  |  |  |  |  |  |  |  |  | N393D |  |  |  |
|  |  |  |  |  |  |  |  |  | T394S |  |  |  |
|  |  |  |  |  |  |  |  |  |  |  |  |  |
| **reverting mutations in R3** |  |  |  |  |  |  |  |  |  |  |  |  |
| **t1** |  |  |  |  |  |  | E98D |  |  |  |  |  |
| **t2** |  |  |  |  |  |  |  |  | D393N |  |  |  |
| **t3** |  |  |  |  |  |  |  |  | T290A |  |  |  |
|  |  |  |  |  |  |  |  |  |  |  | E347N |  |
|  |  |  |  |  |  |  |  |  |  |  | K353Q |  |
|  |  |  |  |  |  |  |  |  |  |  | A354V |  |
|  |  |  |  |  |  |  |  |  | E362G |  |  |  |
|  |  |  |  |  |  |  |  |  |  |  |  |  |
| **forward mutations in R3** | A*24 | A*26 | B*27 | B*0801 | A*24 | A*26 | B*27 | B*0801 | A*24 | A*26 | B*27 | B*0801 |
| **t1** |  |  |  |  |  |  |  |  | K181A/E |  |  |  |
|  |  |  |  |  | K115R/S |  | K115R/S | K115R/S |  |  |  |  |
|  |  |  |  |  |  |  | I133V/T | I133V/T |  |  |  |  |
| **t2** |  |  |  |  | n.a. | n.a. | n.a. | n.a. |  |  |  |  |
| **t3** |  |  |  |  |  | A50T |  |  |  | V84L |  |  |
|  |  |  |  |  |  | G93A | G93A | G93A |  | E86V |  |  |
|  |  |  |  |  |  | L95V/F |  |  |  | N98D |  | N98D |
|  |  |  |  |  |  |  |  | R178N |  | Q113E |  |  |
|  |  |  |  |  |  |  |  |  |  | N129T/K |  | N129T/K |
|  |  |  |  |  |  |  |  |  |  | D132N |  | D132N |
|  |  |  |  |  |  |  |  |  |  | **M134G** |  | M134G |
|  |  |  |  |  |  |  |  |  |  |  | I168L/R | I168L/R |
|  |  |  |  |  |  |  |  |  |  |  | **R169N/G** | R169N/G |
|  |  |  |  |  |  |  |  |  |  |  | D170G | D170G |
|  |  |  |  |  |  |  |  |  |  | Q173K/R | Q173K/R | Q173K/R |

**Table SI** Continued

|  | **Gag** |  |  |  | **Nef** |  |  |  | **Env** |  |  |  |
| --- | --- | --- | --- | --- | --- | --- | --- | --- | --- | --- | --- | --- |
| **forward mutations in R3** | A*24 | A*26 | B*27 | B*0801 | A*24 | A*26 | B*27 | B*0801 | A*24 | A*26 | B*27 | B*0801 |
|  |  |  |  |  |  |  |  |  |  | L178F |  | L178F |
|  |  |  |  |  |  |  |  |  |  |  |  | Y180S |
|  |  |  |  |  |  |  |  |  | **I187V** |  |  |  |
|  |  |  |  |  |  |  |  |  |  |  | **R201V/I/T/M** | **R201V/I/T/M** |
|  |  |  |  |  |  |  |  |  |  |  |  | V217I/T |
|  |  |  |  |  |  |  |  |  |  | Y226F |  |  |
|  |  |  |  |  |  |  |  |  |  | I234L |  | I234L |
|  |  |  |  |  |  |  |  |  |  | K241N/T |  | **K241N/T** |
|  |  |  |  |  |  |  |  |  |  |  |  | T245S |
|  |  |  |  |  |  |  |  |  | D288N |  | D288N | D288N |
|  |  |  |  |  |  |  |  |  |  | **I319M** | I319M | I319M |
|  |  |  |  |  |  |  |  |  | A323T |  | A323T | A323T |
|  |  |  |  |  |  |  |  |  | Y325F/V |  | Y325F/V | Y325F/V |
|  |  |  |  |  |  |  |  |  | T326A |  | T326A | T326A |
|  |  |  |  |  |  |  |  |  | T327S/I |  | T327S/I | T327S/I |
|  |  |  |  |  |  |  |  |  | E329R/D/K |  | E329R/D/K | E329R/D/K |
|  |  |  |  |  |  |  |  |  | **I330V** |  |  |  |
|  |  |  |  |  |  |  |  |  |  |  | I341L | I341L |
|  |  |  |  |  |  |  |  |  |  |  |  | **K345N/E** |
|  |  |  |  |  |  |  |  |  |  | I353V |  | I353V |
|  |  |  |  |  |  |  |  |  |  | K355V/T/S |  | **K355V/T/S** |
|  |  |  |  |  |  |  |  |  |  |  | K364N | **K364N** |
|  |  |  |  |  |  |  |  |  |  |  | T365K | T365K |
|  |  |  |  |  |  |  |  |  | S382T | S382T |  |  |
|  |  |  |  |  |  |  |  |  | Q396P | Q396P |  |  |
|  |  |  |  |  |  |  |  |  |  |  |  | P423Q |
|  |  |  |  |  |  |  |  |  |  |  | M432R | M432R |
|  |  |  |  |  |  |  |  |  |  |  | **R446E/K/S** |  |
|  |  |  |  |  |  |  |  |  |  | I474T |  | I474T |

**Table SI** Continued

|  | **Gag** |  |  |  | **Nef** |  |  |  | **Env** |  |  |  |
| --- | --- | --- | --- | --- | --- | --- | --- | --- | --- | --- | --- | --- |
| **D4** | A*01 | A*03 | B*07 | B*08 | A*01 | A*03 | B*07 | B*08 | A*01 | A*03 | B*07 | B*08 |
|  |  |  |  |  |  | H40Y |  |  |  | E86G |  |  |
|  |  |  |  |  |  |  | R71K |  |  | Y147G |  |  |
|  |  |  |  |  |  | L85V |  |  |  | R148M |  |  |
|  |  |  |  |  |  | **K94M** |  |  |  | W149M |  |  |
|  |  |  |  |  |  | Y115H |  |  |  | S219T |  |  |
|  |  |  |  |  |  |  |  |  |  | D244N |  |  |
|  |  |  |  |  |  |  |  |  |  | E284S |  |  |
|  |  |  |  |  |  |  |  |  |  | **F361V** |  |  |
|  |  |  |  |  |  |  |  |  |  |  | M380T |  |
|  |  |  |  |  |  |  |  |  |  |  |  |  |
| **reverting mutations in R4** |  |  |  |  |  |  |  |  |  |  |  |  |
| **t1** |  |  |  |  |  |  |  |  |  | G86E |  |  |
|  |  |  |  |  |  |  |  |  |  | N244D |  |  |
| **t2** |  |  |  |  |  |  | K71R |  |  |  |  |  |
| **t3** |  |  |  |  |  |  |  |  |  | T219S |  |  |
|  |  |  |  |  |  |  |  |  |  |  | T380M |  |
|  |  |  |  |  |  |  |  |  |  |  |  |  |
| **forward mutations in R4** | A*3604^a^ | A*0201 | B*0801 | B*40 | A*3604^a^ | A*0201 | B*0801 | B*40 | A*3604^a^ | A*0201 | B*0801 | B*40 |
| **t1** |  |  |  |  |  |  |  |  |  | R343K | R343K |  |
|  |  |  |  |  |  |  |  |  |  | A344V/G/E | A344V/G/E |  |
|  |  |  |  |  |  |  |  |  |  | N347H/Q | N347H/Q |  |
|  |  |  |  |  |  |  |  |  |  |  | K364R | K364R |
| **t2** |  |  |  |  |  | **K94E** |  | K94E |  |  | P186Q |  |
|  |  |  |  |  |  |  | E151D | E151D |  |  | I203R |  |
|  |  |  |  |  |  |  |  | S164I/T/N |  |  |  |  |
| **t3** |  |  | L268M |  |  |  | G83A |  |  |  |  | V83L |
|  |  |  |  |  |  |  | E149D | **E149D** |  |  |  | V84E |
|  |  |  |  |  |  |  |  | V182M |  | M134R |  |  |
|  |  |  |  |  |  |  |  |  |  |  | T137D |  |
|  |  |  |  |  |  |  |  |  |  | N142T |  |  |
|  |  |  |  |  |  |  |  |  |  | T144I |  |  |
|  |  |  |  |  |  |  |  |  |  | W149L |  |  |
|  |  |  |  |  |  |  |  |  |  | **V209I** | V209I |  |
|  |  |  |  |  |  |  |  |  |  |  | L297H |  |
|  |  |  |  |  |  |  |  |  |  |  | V301P |  |

**Table SI** Continued

|  | **Gag** |  |  |  | **Nef** |  |  |  | **Env** |  |  |  |
| --- | --- | --- | --- | --- | --- | --- | --- | --- | --- | --- | --- | --- |
| **forward mutations in R4** | A*3604a | A*0201 | B*0801 | B*40 | A*3604a | A*0201 | B*0801 | B*40 | A*3604a | A*0201 | B*0801 | B*40 |
|  |  |  |  |  |  |  |  |  |  |  | I303K |  |
|  |  |  |  |  |  |  |  |  |  |  | T326A |  |
|  |  |  |  |  |  |  |  |  |  |  |  | **E329D** |
|  |  |  |  |  |  |  |  |  |  |  | D333N |  |
|  |  |  |  |  |  |  |  |  |  | K351R | K351R |  |
|  |  |  |  |  |  |  |  |  |  |  | **R358G** | R358G |
|  |  |  |  |  |  |  |  |  |  |  | I474T |  |
|  |  |  |  |  |  |  |  |  |  | K497Q | **K497Q** |  |

**Table SI** Continued

|  | **Gag** |  |  |  | **Nef** |  |  |  | **Env** |  |  |  |
| --- | --- | --- | --- | --- | --- | --- | --- | --- | --- | --- | --- | --- |
| **D5** | A*0201 | A*3201 | B*07 | B*35 | A*0201 | A*3201 | B*07 | B*35 | A*0201 | A*3201 | B*07 | B*35 |
|  |  |  |  |  |  |  | R71K | R71K |  |  |  | N129T |
|  |  |  |  |  |  |  |  | G83A |  |  |  | D132E/N |
|  |  |  |  |  |  |  |  | **L85V** |  |  |  | **V172I** |
|  |  |  |  |  |  |  |  | K105R | V184I |  |  | V184I |
|  |  |  |  |  |  |  |  | R178N |  |  | R261K | **R261K** |
|  |  |  |  |  |  |  |  |  |  |  |  | E278G |
|  |  |  |  |  |  |  |  |  |  |  |  | T292N |
|  |  |  |  |  |  |  |  |  |  |  |  | T326A |
|  |  |  |  |  |  |  |  |  |  |  |  | E329D |
|  |  |  |  |  |  |  |  |  |  |  |  | A344E |
|  |  |  |  |  |  |  |  |  |  |  |  | K345D |
|  |  |  |  |  |  |  |  |  |  |  |  | K355A/D/E |
|  |  |  |  |  |  |  |  |  |  |  | R358K | R358K |
|  |  |  |  |  |  |  |  |  |  |  |  | R425K |
|  |  |  |  |  |  |  |  |  |  | M432R |  | **M432R** |
|  |  |  |  |  |  |  |  |  |  |  |  |  |
| **reverting mutations in R5** |  |  |  |  |  |  |  |  |  |  |  |  |
| **t1** |  |  |  |  |  |  |  |  |  |  |  | T129N |
|  |  |  |  |  |  |  |  |  |  |  |  | E/N132D |
|  |  |  |  |  |  |  |  |  |  |  |  | I184V |
|  |  |  |  |  |  |  |  |  |  |  | K261R | K261R |
|  |  |  |  |  |  |  |  |  |  |  |  | G278E |
|  |  |  |  |  |  |  |  |  |  |  |  | N292T |
|  |  |  |  |  |  |  |  |  |  |  |  | D329E |
|  |  |  |  |  |  |  |  |  |  |  | K358R | K358R |
|  |  |  |  |  |  |  |  |  |  |  |  | K425R |
|  |  |  |  |  |  |  |  |  |  | R432M |  | R432M |
| **t2** |  |  |  |  |  |  | K71R | K71R |  |  |  |  |
| **t3** |  |  |  |  |  |  |  |  |  |  |  |  |
|  |  |  |  |  |  |  |  |  |  |  |  |  |
| **forward mutations in R5** | A*0207 | A*0207 | B*0801 | B*27 | A*0207 | A*0207 | B*0801 | B*27 | A*0207 | A*0207 | B*0801 | B*27 |
| **t1** |  |  |  |  |  |  |  |  |  |  | N163K/M |  |
|  |  |  |  |  |  |  |  |  |  |  | I164N |  |
|  |  |  |  |  |  |  |  |  |  |  | **R169K/N** | **R169K/N** |
|  |  |  |  |  |  |  |  |  |  |  | V172M/T | V172M/T |

**Table SI** Continued

|  | **Gag** |  |  |  | **Nef** |  |  |  | **Env** |  |  |  |
| --- | --- | --- | --- | --- | --- | --- | --- | --- | --- | --- | --- | --- |
| **forward mutations in R5** | A*0207 | A*0207 | B*0801 | B*27 | A*0207 | A*0207 | B*0801 | B*27 | A*0207 | A*0207 | B*0801 | B*27 |
|  |  |  |  |  |  |  |  |  |  |  | E284K | E284K |
| **t2** |  |  |  |  |  |  | **K94E/Q** | K94E/Q |  |  | R150K | **R150K** |
|  |  |  |  |  |  |  | **K105S** | K105S |  |  |  |  |
|  |  |  |  |  |  |  | P150Q |  |  |  |  |  |
| **t3** |  |  | **R264Q** | **R264Q** |  |  |  |  |  |  | D170G | D170G |
|  |  |  |  |  |  |  |  |  |  |  | Q173K | Q173K |
|  |  |  |  |  |  |  |  |  |  |  | Y200F | Y200F |
|  |  |  |  |  |  |  |  |  |  |  | **R201V** | **R201V** |
|  |  |  |  |  |  |  |  |  |  |  | V209R | V209R |
|  |  |  |  |  |  |  |  |  |  |  | V217I |  |
|  |  |  |  |  |  |  |  |  |  |  | T292I |  |
|  |  |  |  |  |  |  |  |  |  |  | E299N/Q |  |
|  |  |  |  |  |  |  |  |  |  |  | K314R | K314R |
|  |  |  |  |  |  |  |  |  |  |  | H217T | H217T |
|  |  |  |  |  |  |  |  |  |  |  | Y325L | Y325L |
|  |  |  |  |  |  |  |  |  |  |  | T327S | T327S |
|  |  |  |  |  |  |  |  |  |  |  | G328R | G328R |
|  |  |  |  |  |  |  |  |  |  |  | E329R | E329R |
|  |  |  |  |  |  |  |  |  |  |  | **K355V** |  |
|  |  |  |  |  |  |  |  |  |  |  | **R446E** | **R446E** |
|  |  |  |  |  |  |  |  |  |  |  | G478A |  |

^a^Epitopes for subtypes A*2301, A*3604 and B*49 were not available in the Los Alamos database and therefore only the other HLA epitopes were used for prediction of epitopes. For reference, sequences were aligned to the HIV-1 subtype B consensus sequence. All sequences were derived from isolated clonal HIV-1 variants; t1: 2-3 weeks after transmission, t2: 9-22 months after transmission and, t3: >4.5 after transmission. Depicted in bold are mutations at anchor residue positions.
