# Supplementary figures and images for: Molecular Evolution of Human Immunodeficiency Virus Type 1 upon Transmission between Human Leukocyte Antigen Disparate Donor-Recipient Pairs
Source: PLoS One. 2008 Jun 18;3(6):e2422. doi: 10.1371/journal.pone.0002422 (PMC2409968; doi:10.1371/journal.pone.0002422)

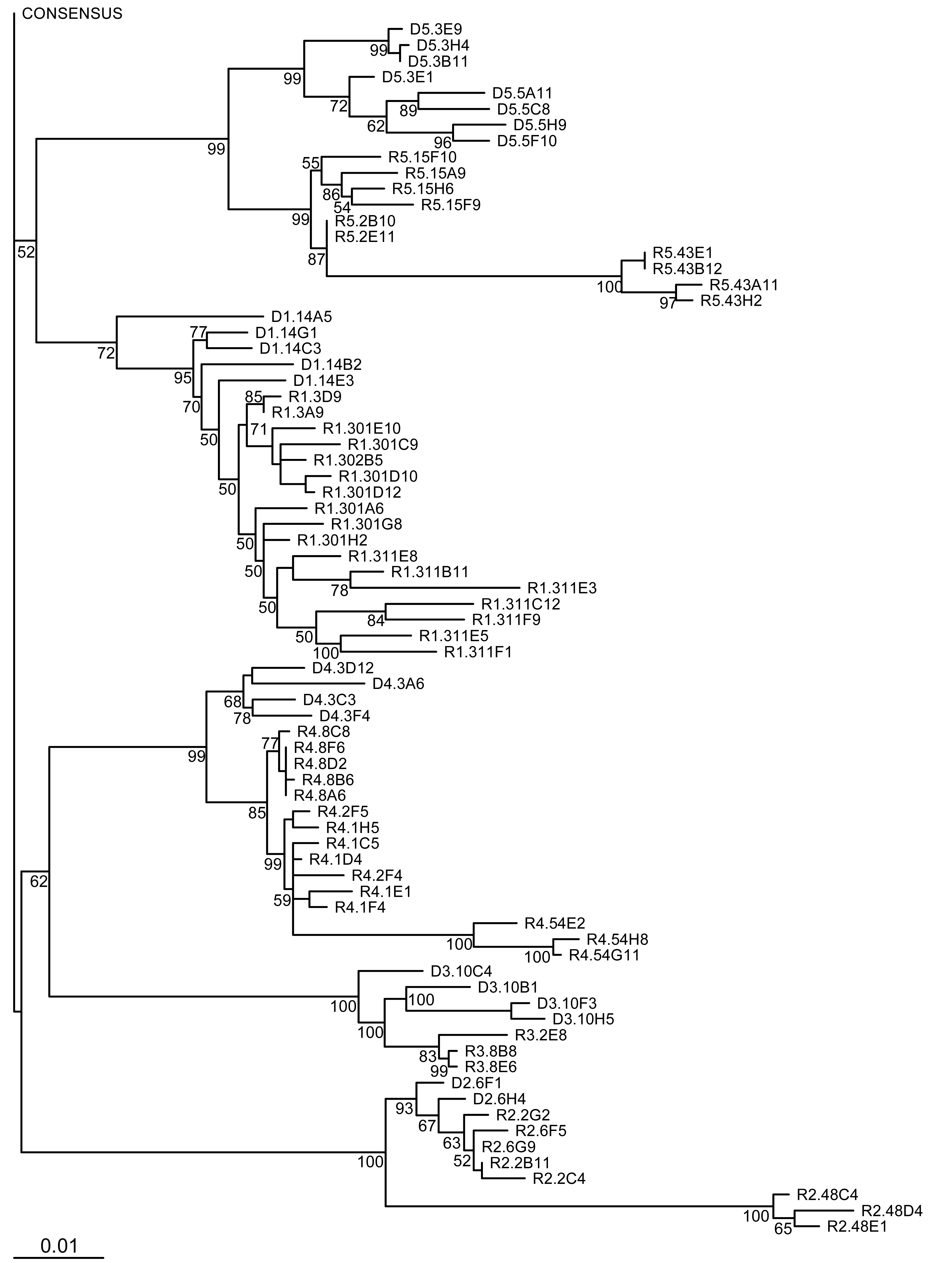

Supplement: Figure S1 — Phylogenetic analysis of env sequences of clonal HIV-1 variants isolated from donors (D1-5) and recipients (R1-5) involved in homosexual HIV-1 transmission. Shown is a maximum likelihood tree with bootstrap values obtained from neighbor joining analysis. Bootstrap values are given and show that HIV-1 variants from donors and recipients are related. (0.35 MB TIF) [file pone.0002422.s002.tif]
